# General control nonderepressible 2 (GCN2) as a therapeutic target in age-related diseases

**DOI:** 10.3389/fragi.2024.1447370

**Published:** 2024-09-10

**Authors:** Ozlem Altintas, Michael R. MacArthur

**Affiliations:** ^1^ Department of Health Sciences and Technology, ETH Zurich, Zurich, Switzerland; ^2^ Lewis-Sigler Institute for Integrative Genomics, Princeton University, Princeton, NJ, United States

**Keywords:** GCN2, ISR, amino acids, aging, gerotherapeutic, geroprotection, cancer

## Abstract

The function of General Control Nonderepressible 2 (GCN2), an evolutionary-conserved component of the integrated stress response (ISR), has been well-documented across organisms from yeast to mammals. Recently GCN2 has also gained attention for its role in health and disease states. In this review, we provide a brief overview of GCN2, including its structure, activation mechanisms and interacting partners, and explore its potential significance as a therapeutic target in various age-related diseases including neurodegeneration, inflammatory disorders and cancer. Finally, we summarize the barriers to effectively targeting GCN2 for the treatment of disease and to promote a healthier aging process.

## Introduction

GCN2 was first identified in yeast as a serine/threonine kinase and later recognized as the homolog of mouse and human eukaryotic translation initiation factor 2 alpha kinase 4 (*EIF2AK4*). It functions as an amino acid sensor and serves to activate the integrated stress response (ISR) upon amino acid deprivation. Proper ISR activation is essential for cells to make “live or die” decisions and maintain organismal homeostasis under stress, and GCN2 plays a central role in this system.

**FIGURE 1:**
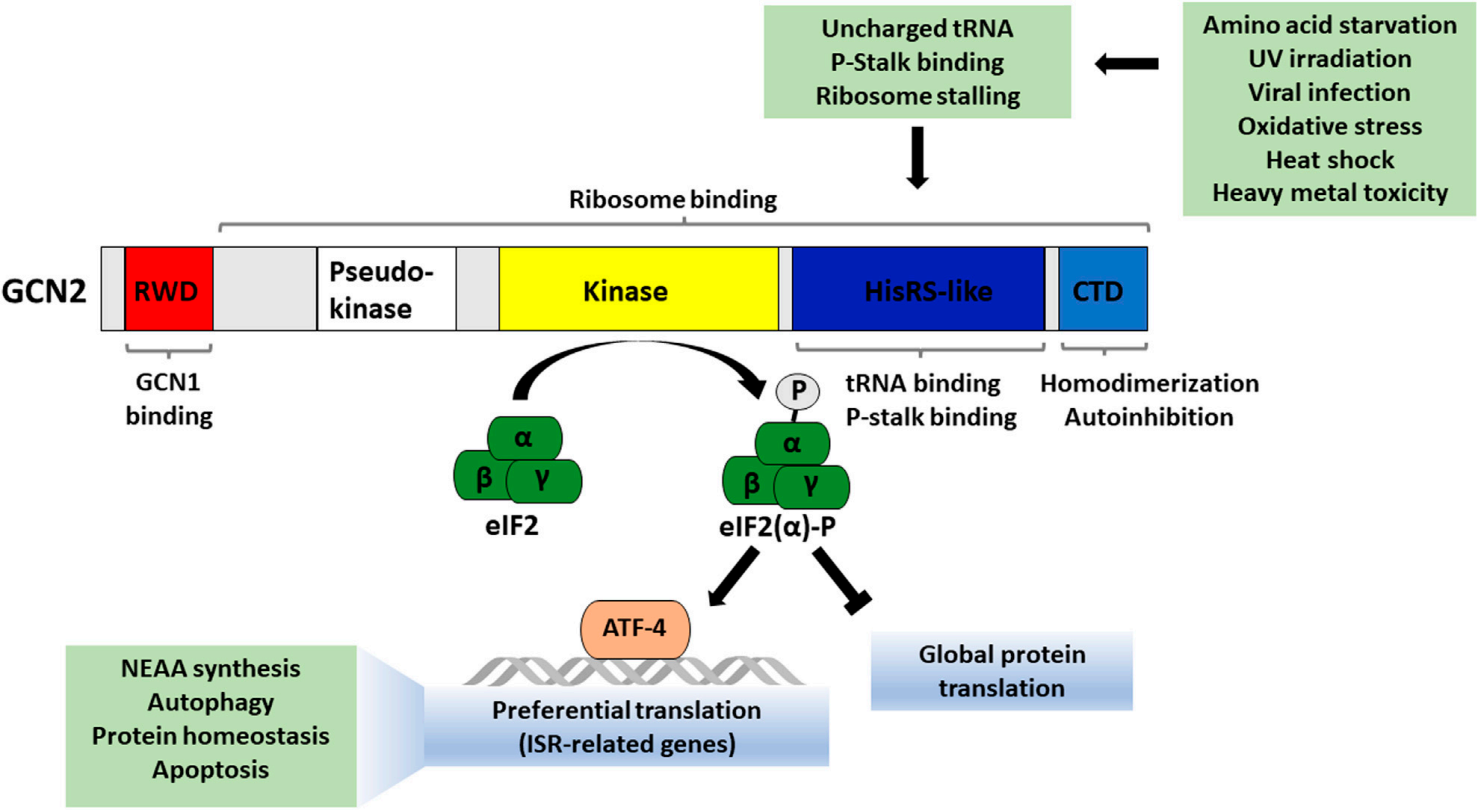
Overview of GCN2 protein domains and factors that activate GCN2. Active GCN2 phosphorylates the α subunit of eukaryotic translation initiation factor 2 which reduces global protein translation and induces selective stress-adaptive gene transcription, with targets including ATF4.

More than two decades ago, GCN2’s role in nutrient sensing was first reported in a screen for genes functioning in the general amino acid control pathway in budding yeast *Saccharomyces cerevisiae* (Hinnebusch, 1985). The activation of GCN2 under amino acid starvation has been well studied, particularly in yeast. When levels of a specific amino acid are limiting, deacylated (“uncharged”) levels of the corresponding tRNA increase. Uncharged tRNAs directly bind to GCN2 on its histidyl-tRNA synthetase-like (HisRS) domain and thereby convert GCN2 from an autoinhibited state to a functionally active state by driving homodimerization and autophosphorylation (Diallinas and Thireos, 1994; Wek et al., 1995; Narasimhan et al., 2004; Gallinetti et al., 2013) (Figure 1). Active GCN2 phosphorylates the alpha subunit of the translation initiation factor eIF2 (EIF2A) at serine 51 (Lu et al., 1999; Holcik and Sonenberg, 2005; Ravindran et al., 2014). This leads to a decrease in global protein translation while allowing for preferential translation of specific stress-responsive transcripts, including activating transcription factor 4 (ATF4), which can push a cell towards either survival or regulated cell death depending on the severity of the stress stimulus (Pakos-Zebrucka et al., 2016; Costa-Mattioli and Walter, 2020). The ISR pathway also comprises a negative feedback component, including ATF4-driven expression of machinery to dephosphorylate eIF2α, allowing a return to normal cellular function once the stressor is overcome. The ISR plays a major role in health and disease states and tends to become dysregulated with aging. Additionally, mild activation of GCN2 via dietary protein dilution or individual amino acid restriction has been shown to extend lifespan in preclinical models.

As populations worldwide are rapidly aging, it is critical to identify strategies to extend healthspan and prevent or delay age-related diseases. Here, we review the role of GCN2 in the ISR and its potential as a therapeutic target in age-related diseases.

## Structure and activation of GCN2

GCN2 was first identified as an amino acid sensor, and amino acid deprivation is its canonical activating stimulus. However, it has also been shown that other stress stimuli can activate GCN2, including UV irradiation, oxidative stress, ribosome collisions, ribosomal p-stalk binding and proteasome inhibition (Deng et al., 2002; Jiang and Wek, 2005; Anda et al., 2017; Harding et al., 2019; Inglis et al., 2019; Wu et al., 2020).

GCN2 consists of five conserved domains, including an N-terminal RWD domain (named due to its presence in RING finger and WD repeat containing proteins and DEAD-like helicases), pseudokinase domain, kinase domain, HisRS-like domain (with sequence similarity to histidyl-tRNA synthetase) and C-terminal domain (CTD) (Figure 1). Under conditions of amino acid sufficiency, GCN2 is in an inactive dimeric form, and the physical interaction between its kinase domain, HisRS-like domain, and CTD prevents its activation (Lageix et al., 2015). Binding of uncharged tRNAs is required for sensing amino acid deprivation by GCN2. Upon binding of uncharged tRNAs to its HisRS-like domain, allosteric changes in GCN2’s structure cause the loss of autoinhibitory contacts between its domains. GCN2 gets activated via autophosphorylation (Threonine-882 and -887 in *S. cerevisiae*, Threonine-898 and -903 in mouse, and Threonine-899 and -904 in human) in the activation loop of its kinase domain (Romano et al., 1998; Sood et al., 2000; Cherkasova et al., 2010). Finally, active GCN2 phosphorylates its substrate, eIF2α (Qiu et al., 2001; Padyana et al., 2005; Gárriz et al., 2009). Phosphorylated eIF2α forms a complex with eIF2B, preventing its function as a guanine nucleotide exchange factor. When recycling of GDP for GTP on eIF2 is inhibited, the ternary complex cannot form, and translation cannot initiate. This set of events leads to a decrease in global translation. At the same time, there is also an increase in the translation of a specific set of stress-responsive transcripts, including the transcription factor ATF4 (Blais et al., 2004).

The full crystal structure of GCN2 in inactive and active states and in complex structure with its interacting partners (GCN1, GCN20, and ribosomal components) have yet to be elucidated. Our current knowledge of GCN2 structure is limited to its kinase domain and CTD (Padyana et al., 2005; He et al., 2014; Maia de Oliveira et al., 2020). Further studies on the complete crystal structure of GCN2 will help to better understand the structural regulation of GCN2 which will eventually aid in the development of structure-based drugs to modulate GCN2 activity.

## Interacting partners of GCN2

Several conserved proteins interact with GCN2 directly or indirectly to modulate its activity. Gcn1 (general control non-derepressible 1) and Gcn20 (an ATP-binding cassette family protein) are important for the activation of yeast Gcn2 during amino acid starvation *in vivo* but not required for GCN2 kinase function *in vitro* (Hinnebusch, 1985; Marton et al., 1993; Vazquez de Aldana et al., 1995). Gcn1 also has binding domains for ribosome components and Gcn20, both of which are important for Gcn2 activation (Vazquez de Aldana et al., 1995; Marton et al., 1997; Pochopien et al., 2021). The Gcn1/Gcn20 protein complex binds to the GCN2 N-terminus, and this binding is required for efficient GCN2 activation *in vivo* (Garcia-Barrio et al., 2000). GCN1 is subject to negative regulation by yeast Yih1 (mammalian IMPACT), which prevents GCN1 binding to GCN2, thus indirectly inhibiting GCN2 (Pereira et al., 2005). GCN1 is highly conserved across yeast, *Arabidopsis*, *Drosophila* and mammals (Marton et al., 1993; Izquierdo et al., 2018) with multiple examples of cross-species interaction between GCN1 and GCN2 homologs (Garcia-Barrio et al., 2000). While Gcn20 does not interact with the ribosome or Gcn2 directly, it supports the interaction between Gcn1 and Gcn2 and tethering of the complex to the ribosome. Within the complex, Gcn1 directly binds to the RWD domain of Gcn2 and functions as a chaperone to transfer uncharged tRNA from the ribosomal A-site to the HisRS-like domain of Gcn2 upon amino acid starvation, thus facilitating Gcn2 activation (Sattlegger and Hinnebusch, 2000; 2005).

GCN2 targets multiple downstream effectors to control translation initiation. In addition to eIF2α (yeast Sui2), GCN2 also phosphorylates the beta subunit of the eIF2 complex (yeast Sui3) upon amino acid deprivation. This promotes association with the translation initiation factor eIF5 and restricts recycling of the eIF2 ternary complex (Dokládal et al., 2021). Additionally, GCN2 engages in an inhibitory feedback loop through which GCN20 phosphorylation weakens GCN1-GCN20 complex formation and suppresses activation of GCN2 by uncharged tRNAs (Dokládal et al., 2021). These multiple interaction partners represent the various layers of control in the GCN2 signaling pathway with targeted disruption of these interactions representing a promising strategy to control GCN2 activation.

## GCN2 in longevity

GCN2 has been implicated in the control of lifespan across multiple species. In *Saccharomyces cerevisiae* Gcn2 activity is required for lifespan extension by amino acid restriction (Wu et al., 2013). Another study in yeast showed that Gcn2 activation upon tRNA overexpression suppresses global translation and extends the lifespan of young cells in a Gcn4 (yeast ATF4)-dependent manner (Hu et al., 2018). In *Drosophila melanogaster* GCN2/ATF4 regulate the induction of the translational repressor 4E-BP to extend lifespan upon ER stress and nutrient restriction (Kang et al., 2017). Tissue-specific knockdown of GCN2 in the gut and fat body, two energy-sensing organs in *Drosophila* which are counterparts of mammalian liver and adipose tissue, abolishes the beneficial effect of dietary amino acid restriction on lifespan (Kim et al., 2020).

In *Caenorhabditis elegans*, deletion or RNAi knockdown of *gcn-2* produces minimal phenotype in the basal unstressed condition in terms of lifespan, growth, development, movement, and fecundity (Rousakis et al., 2013). However, lifespan extension in *eat-2* and *let-363* mutants were lost upon *gcn-2* knockout, suggesting a role for *gcn-2* in lifespan extension by dietary restriction and TOR inhibition (Rousakis et al., 2013). Deletion of the *gcn-2* negative regulator *impt-1* resulted in ISR activation and lifespan extension in *C. elegans* (Ferraz et al., 2016). Lifespan extension by *impt-1* deletion was dependent on both *gcn-2* and the *C. elegans* ATF4 homolog *atf-5* (Ferraz et al., 2016).

Data across species demonstrate that activation of GCN2 by multiple stimuli, including amino acid restriction, is associated with increased lifespan. Multiple mechanisms may contribute downstream of GCN2 activation. First, interventions that reduce global protein translation are associated with extended lifespan (Hansen et al., 2007). Second, increased selective translation of stress-responsive factors, including ATF4, can drive geroprotective molecular programs such as increased autophagy (Li et al., 2014; Statzer et al., 2022). This combination of reduced global translation and induction of pro-survival pathways during perceived nutrient deprivation provides the conditions for the organism to save energy and ensure survival requirements are optimally met. While the causal evidence in non-mammalian systems is robust, data on the role of GCN2 in mammalian aging is currently only associative. Evidence is still lacking on whether GCN2 signaling is altered in aging, or whether GCN2 is required for lifespan extension by nutrient restriction in mammals. Further, whether direct modulation of GCN2 activity or levels impacts the mammalian aging process also remains an important open question.

## GCN2 in healthy aging

Aging is a complex process that drives changes across physiologic and molecular domains, collectively giving rise to pathological conditions that increase an individual’s susceptibility to age-related diseases. A properly functioning ISR maintains a delicate balance between health and disease in organisms across the lifespan. GCN2 contributes to the critical regulation by the ISR to drive cell survival or death and has been associated with inflammatory, metabolic, neurological and metastatic diseases. Despite substantial progress, the potential therapeutic applications of GCN2 remain an ongoing and dynamic field of research.

GCN2 plays a significant role in the immune system and is associated with various diseases related to immune function as reviewed in Xia et al. (2018). GCN2 is reported to suppress the activation of the inflammasome and control inflammation in the intestine (Ravindran et al., 2016). GCN2-deficient mice display hypersensitivity to DSS-induced colitis with increased weight loss, enhanced inflammation, Th17 response, colonic shortening, and impairment of the epithelial barrier. Conversely, GCN2 activation by amino acid restriction significantly protects against DSS colitis by inhibiting reactive oxygen species-mediated inflammasome activation, suppressing pathogenic Th17 response and promoting autophagy to reduce inflammation (Ravindran et al., 2016; Revelo et al., 2016). Tissue-specific GCN2 deletion in intestinal epithelial cells and CD11c+ antigen-presenting cells (APCs) also leads to increased intestinal inflammation, weight loss and colon shortening in mice. These data in colitis models support GCN2 as a potential target in intestinal inflammatory diseases with therapeutic strategies including activation by either small molecules or amino acid-restricted diets.

GCN2 supports adaptation to amino acid stress by the immune system, while GCN2 loss renders the immune system more sensitive to the cytotoxic effects of amino acid deprivation, leading to enhanced lymphocyte loss (Bunpo et al., 2010). Under basal conditions, the immune system prevents autoimmunity by clearing apoptotic cells and driving immune tolerance toward self-antigens. Myeloid GCN2 deletion in an autoimmunity-prone mouse model suppresses the development of tolerance and increases autoimmunity, renal pathology and overall mortality (Ravishankar et al., 2015). This is driven by a lack of apoptotic clearance as GCN2 null liver macrophages are unable to clear damaged red blood cells and recycle iron upon hemolytic stress (Toboz et al., 2022). Mechanistically, apoptotic cells activate the tryptophan-catabolizing enzyme IDO1 in macrophages which signals to GCN2 to regulate cytokine production, increasing anti-inflammatory IL-10 responses while decreasing pro-inflammatory IL-12 (Ravishankar et al., 2015). GCN2 is required for IDO1 produced by dendritic cells to signal and suppress proliferation in T-cells (Munn et al., 2005; Baban et al., 2009; Sharma et al., 2009). GCN2 activation in mice suppresses pro-inflammatory cues and decreases the recruitment of macrophages to the kidney during glomerular inflammation (Chaudhary et al., 2015), while the inhibition of autophagy or knockout of IDO1 or GCN2 in mice exacerbates nephritis to fatal end-stage renal failure. Conversely, increasing kidney IDO1 activity or treating mice with a GCN2 agonist drives autophagy and protects mice from nephritic kidney damage.

Many studies have also implicated GCN2 activity in cancer, and a growing body of evidence supports modulating GCN2 activity as a potential cancer therapeutic strategy (Gold and Masson, 2022). A common characteristic of the tumor microenvironment is limited nutrient availability. As cells require an increased supply of nutrients due to enhanced anabolism, the consumption of available nutrients can rapidly drive nutrient deprivation within tumors (DeBerardinis et al., 2008). To deal with the nutrient stress, some cancer cells activate GCN2, which drives adaptations, including increased expression of amino acid transporters and non-essential amino acid synthetic enzymes (Wang et al., 2013; Cordova et al., 2022). For example, GCN2 drives the expression of asparagine synthetase (ASNS) via eIF2a/ATF4, which is required for cancer cell survival upon asparaginase treatment. Co-treatment with asparaginase and GCN2 inhibition or deletion synergize with asparaginase against multiple cancer cell types (Ye et al., 2010; Wang et al., 2013; Nakamura et al., 2018). Similarly, hepatocellular carcinoma cells become auxotrophic for arginine due to suppressed urea cycle and are sensitive to GCN2 inhibition or deletion due to a requirement for GCN2-driven expression of the transporter SLC7A1 (Missiaen et al., 2022). GCN2 also promotes VEGF-driven angiogenesis, supporting tumor cell growth and survival by increasing nutrient supply (Ye et al., 2010; Longchamp et al., 2018; Zhang et al., 2021).

Autophagy is an important process for cancer cell survival as it recycles proteins and other macromolecules during nutrient deprivation and improves the resistance of cancer cells to stress conditions that normally lead to cell death including hypoxia (Galluzzi et al., 2015). Under leucine deprivation, ATF4 drives transcription of autophagy genes in a GCN2-dependent manner, supporting cell survival (B’chir et al., 2013). These findings all support the suppression of GCN2 activity as a promising therapeutic strategy to suppress adaptive amino acid transport/synthesis, angiogenesis, and autophagy in cancer cells.

Cancer therapy-induced cardiotoxicity caused by chemotherapy including doxorubicin (Dox) is a significant challenge in cancer treatment and is strongly linked to morbidity and mortality (Carver et al., 2007). Dox-treated GCN2 knockout mice display less myocardial fibrosis, contractile dysfunction, cardiomyocyte death, and oxidative stress as compared to wildtype mice (Lu et al., 2014; Wang et al., 2018). On the other hand, GCN2 knockout mice were significantly more susceptible to hepatotoxicity during asparaginase treatment, showing increased hepatic oxidative stress and inflammation (Wilson et al., 2013). These findings support modulation of GCN2 activity not only for treating cancer but also for attenuating undesired side effects of specific cancer treatments, although the directionality of effects may vary depending on the specific therapeutic.

In contrast to the potential therapeutic benefits of GCN2 inhibition or deletion in cancer, active GCN2 is required for a proper response to infection (Philips and Khan, 2021; Tomé, 2021; Zhao et al., 2023). A requirement for GCN2 in the inflammatory response to lipopolysaccharide (LPS) has been shown *in vitro* and *in vivo* (Liu et al., 2014). Under low tryptophan conditions, macrophages require active GCN2 for LPS-driven cytokine production, whereas macrophages lacking GCN2 show reduced cytokine levels upon LPS exposure. Similarly, monocytic lineage specific GCN2 knockout mice lack the ability to maximally induce cytokines in response to an LPS challenge (Liu et al., 2014).

GCN2 also has a restrictive effect against viral infections. The silencing of GCN2 results in increased HIV-1 infection and GCN2 loss of function mutant mice display increased susceptibility to viral infection (Pino et al., 2012; Won et al., 2012; Cosnefroy et al., 2013; Jaspart et al., 2017). Additionally, GCN2-deficient mice show increased viral titers in the brain after infection with Sindbis Virus (SV) (Berlanga et al., 2006). The blocking of early viral SV RNA translation by GCN2 inhibits SV replication, pointing out another important function of GCN2 in the cellular response to viruses. Upon yellow fever vaccination, activated GCN2 programs dendritic cells to promote stress granule formation and autophagy, and also increases antigen presentation to CD4^+^ and CD8^+^ T cells (Ravindran et al., 2014; Battu et al., 2017). Interestingly, RNA-sequencing data using CCD841 cells shows that GCN2 promotes the expression of angiotensin-converting enzyme 2 (ACE2), a primary receptor for SARS-CoV-2, upon leucine deprivation, while GCN2 knockdown decreases its expression (Hu et al., 2022). Thus, inhibition of GCN2 might also be an antiviral approach against specific viruses the require ACE2 for cell entry, including COVID-19. It is also important to note that GCN2 activation might be required for the survival of some parasites in the host. A parasite, *Toxoplasma gondii*, consumes arginine within its host cell, which in turn leads to GCN2 activation to provide additional arginine to the infected cells (Augusto et al., 2019). GCN2 deletion reduces arginine levels, suppressing parasite proliferation. These findings support the requirement for GCN2 activation to mount a proper inflammatory response upon bacterial or viral infection and also demonstrate specific viral or parasitic infections where GCN2 inhibition may be beneficial (Afroz et al., 2020).

Gcn2 is highly expressed in the hippocampus and functions in long-term memory formation, synaptic plasticity, and learning, while GCN2 knockout mice display impaired hippocampus-dependent memory and learning (Costa-Mattioli et al., 2005; Ohno, 2014). In APP/PS1 Alzheimer`s disease (AD) model mice, GCN2 deletion alleviates defects in synaptic plasticity and memory (Ma et al., 2013). Interestingly, in another AD mouse model (5XFAD), GCN2 knockout causes no improvements in memory decline, potentially due to compensatory PERK overactivation in the brains (Devi and Ohno, 2013). Considering the differences in the genetic background of model animals, the role of GCN2 in memory decline needs to be further investigated.

Some mutations or SNPs in the gene encoding human GCN2, *EIF2AK4*, have been associated with specific diseases. Loss of function mutations in human GCN2 or decreased GCN2 expression are robustly associated with various types of pulmonary hypertension (Eyries et al., 2014; Eichstaedt et al., 2016; Hadinnapola et al., 2017; Nossent et al., 2018; Manaud et al., 2020; Chen et al., 2021; English et al., 2022; Emanuelli et al., 2024). A genome-wide association study in a Japanese population revealed a risk allele, EIF2AK4 SNP rs2250402, associated with type 2 diabetes mellitus in humans (Yasuda et al., 2008; Kanno et al., 2020). A follow-up study revealed that this allele was associated with reduced insulin secretion, and that mice lacking GCN2 had reduced pancreatic beta cell mass when fed a high fat diet (Kanno et al., 2020). Further genomic studies are also needed in different ethnic groups to validate the generalizability of these risk variants in EIF2AK4 across populations.

GCN2 also plays an important role in critical organ function. GCN2 deletion mitigates muscle atrophy induced by denervation while its overexpression exacerbates it (Guo et al., 2018). Additionally, patients suffering from myocardial ischemia and hypoxia-reperfusion display elevated levels of GCN2 and GCN2 expression is elevated in an oxygen-glucose deprivation/reoxygenation (OGD/R) model while its knockdown decreases oxidative stress, inflammation and apoptosis in embryonic rat cardiomyocytes upon OGD/R (Pu et al., 2019; Zhang et al., 2022). GCN2 deletion or GCN2iB treatment in diabetic mice both improves cardiac function by decreasing oxidative stress and lipotoxicity (Feng et al., 2019; Yuan et al., 2022). In this case, strategies reducing the activity of GCN2 may be a therapeutic strategy in treating muscle atrophy or OGD/R in cardiac muscle. Similarly, GCN2 knockout mice had significantly lower serum urea and creatinine in hepatic and renal ischemia-reperfusion models, however they did not gain additional protection from a tryptophan-deficient diet (Peng et al., 2012). GCN2 affects the production of collagen type I by hepatic satellite cells and protects mice from liver injury and fibrosis (Arriazu et al., 2013). It also regulates hepatic gluconeogenesis, as liver-specific GCN2 KO mice show a deficiency in maintaining glucose homeostasis during fasting (Xu et al., 2013). While the role of GCN2 in disease is complex, the fine-tuning of the activity or expression level of GCN2 protein under different physiological conditions may represent a promising therapeutic approach for multiple diseases.

## Discussion

Aging is a remarkably complex process characterized by dramatically increased susceptibility to a variety of diseases. GCN2 has been studied across various diseases models, including immune disorders, cancer, infection, and memory loss which are all age-associated disorders or exacerbated with aging. The involvement of GCN2 in such a wide variety of age-related diseases underscores the importance of a more comprehensive understanding of this critical nutrient-sensing kinase. Such understanding is crucial for the advancement of novel and efficient therapeutic candidates, aiming to mitigate disease symptoms and contribute to a healthier aging experience.

There are some challenges in drug development to regulate GCN2 activity with direct physical interaction. Firstly, the complete crystal structure of human GCN2 has yet to be elucidated. The 3D structure of GCN2 changes significantly as it converts from inactive to active states. As an example of the challenge this present, one of the most widely used GCN2 inhibitors was recently shown to act as a GCN2 *activator* at certain doses (Carlson et al., 2023). A detailed understanding of the state-specific structure of the kinase and its domains will help researchers design drugs that can specifically target GCN2, potentially even when it is in certain states, to enhance or suppress its activity. Additionally, human GCN2 has 3 isoforms. The function of each isoform, its tissue-specific functionality, and its working mechanism in different stress conditions might differ.

As the RWD domain in the N terminal region of GCN2 interacts with GCN1, targeting this domain may be an effective way to control GCN2 activation. Blocking this domain with specific small molecules might hinder its interaction with GCN1 and render the kinase inactive. Targeting GCN2 activity indirectly by regulating the function, structure, or availability of its interacting molecules may also be possible. One way to regulate GCN2 activation might be achieved through blocking the binding of ribosomal P-stalk (Harding et al., 2019; Inglis et al., 2019), for example, by targeting the C-terminus of P-stalk proteins or domain II of uL10, which were shown to be the regions that interact with GCN2 to activate it *in vitro*.

During periods of nutrient scarcity, GCN2 establishes a connection between nutrient sensing and longevity by globally inhibiting translation and selectively activating stress responses, preserving homeostasis. This mechanism has demonstrated a robust ability to enhance lifespan and healthspan in invertebrates. However, GCN2’s impact on aging in more complex organisms remains underexplored. Despite the uncertain role of GCN2 in core mammalian aging processes, it is clear that GCN2 plays a critical role in multiple diseases of aging in mammals and holds great promise as a therapeutic target. Further work is needed to develop therapeutic strategies that allow for the targeted modulation of GCN2 levels and activity to treat diseases of aging.
